# Etoposide Incorporated into Camel Milk Phospholipids Liposomes Shows Increased Activity against Fibrosarcoma in a Mouse Model

**DOI:** 10.1155/2015/743051

**Published:** 2015-03-02

**Authors:** Hamzah M. Maswadeh, Ahmad N. Aljarbou, Mohammed S. Alorainy, Mansour S. Alsharidah, Masood A. Khan

**Affiliations:** ^1^Department of Pharmaceutics, College of Pharmacy, Qassim University, Buraidah 51412, Saudi Arabia; ^2^Department of Pharmacology & Therapeutics, College of Medicine, Qassim University, Buraydah, Saudi Arabia; ^3^Department of Physiology, College of Medicine, Qassim University, Buraydah, Saudi Arabia; ^4^Basic Health Sciences, College of Applied Medical Sciences, Qassim University, Buraydah, Saudi Arabia

## Abstract

Phospholipids were isolated from camel milk and identified by using high performance liquid chromatography and gas chromatography-mass spectrometry (GC/MS). Anticancer drug etoposide (ETP) was entrapped in liposomes, prepared from camel milk phospholipids, to determine its activity against fibrosarcoma in a murine model. Fibrosarcoma was induced in mice by injecting benzopyrene (BAP) and tumor-bearing mice were treated with various formulations of etoposide, including etoposide entrapped camel milk phospholipids liposomes (ETP-Cam-liposomes) and etoposide-loaded DPPC-liposomes (ETP-DPPC-liposomes). The tumor-bearing mice treated with ETP-Cam-liposomes showed slow progression of tumors and increased survival compared to free ETP or ETP-DPPC-liposomes. These results suggest that ETP-Cam-liposomes may prove to be a better drug delivery system for anticancer drugs.

## 1. Introduction

Cancer is a growing health concern around the world due to changes in environmental conditions and lifestyle. The existing treatment approaches have not been able to effectively deal with cancer and, therefore, continuing efforts are ongoing to explore novel strategies for cancer treatment.

Most clinical regimens comprise multiple non-cross-resistant anticancer agents [1]. DNA-modifying agents such as anthracycline-based topoisomerase II inhibitors and platinum-based drugs have been explored alone or in combination against ovarian, advanced breast cancer, and endometrial carcinoma [2–10]. Etoposide, a topoisomerase II inhibitor, is used in the chemotherapy of various types of cancers, including lymphoma, lung cancer, leukemia, and Kaposi's sarcoma. Cancerous cells rely on more topoisomerase II than healthy cells; thus targeting this enzyme is one of the important strategies to control the multiplication of cancerous cells. Etoposide makes a ternary complex with topoisomerase II and DNA, causing DNA breakage and thus apoptosis [11]. Etoposide shows dose-limiting hematologic and gastrointestinal toxicity. Liposomal formulations of anticancer drugs, including etoposide, have been explored to increase their activity and to reduce their toxicity [12, 13].

Liposomes make an excellent drug delivery system as they are both biocompatible and biodegradable. They incorporate both lipophilic and hydrophilic drugs. Drug delivery systems have been shown to reverse the chances of drug resistance in cancer cells [14]. Moreover, liposomized anticancer formulations can circumvent drug efflux and achieve adequate drug concentrations in the target cells to enable tumor killing, because liposome-mediated disposition interacts well with drug efflux mediated by p-glycoprotein transporters [14, 15].

Evidently, if the liposomes are to be used for targeting extra-RES tissues, a key issue is to reduce the rate of uptake by the RES to enable them to remain in the circulation longer. The effect of particle size in favor of small vesicles was recognized early [16]. It has been noted previously that vesicles with a mean diameter of 100 nm exhibit longer circulation times than smaller or larger vesicles with the same composition [17].

Camel milk is an important source of proteins and is widely exploited for human health [18]. Phospholipids (PLs) are the main constituents of the milk fat globule membrane (MFGM). MFGM is trilaminar, mainly consisting of proteins, surrounding the intracellular neutral lipids. This inner part is covered by a bilayer membrane derived from the secretory cell apical plasma membrane [19]. PLs are mainly located in the outer leaflet and are organized as a liquid-disordered phase coexisting with a liquid-ordered phase (also called a lipid raft) and the latter is rich in sphingomyelin and cholesterol [20, 21].

The aim of the present work was to extract and analyze the major PLs present in camel milk by using HPLC and GC/MS. The isolated total camel milk phospholipids were used to prepare SUVs liposomes and the latter were incorporated with etoposide. The* in vivo* activity of various etoposide formulations prepared in this study was tested against experimental fibrosarcoma in a murine model.

## 2. Materials and Methods

### 2.1. Materials

1,2-Dipalmitoyl-sn-glycero-3-phosphatidylcholine (DPPC), synthetic soya phosphatidylcholine (PC), phosphatidylethanolamine (PE), phosphatidylinositol (PI), and lysophosphatidylcholine (LPC) standards were obtained from Avanti Polar Lipids, Inc. (Alabaster, AL, USA). Silica gel-60 and precoated thin layer chromatography (TLC) plates, triethyamine, acetonitrile, methanol, chloroform, and phosphoric acid of HPLC grade were purchased from Merck (Germany). Etoposide was obtained from Tocris Bioscience (UK). Sephadex G-75 was obtained from Fisher Scientific (Loughborough, UK). Camel milk was collected from a six-year-old red camel two months postdelivery lactation period from Aljarbou farm in Qassim, Saudi Arabia.

### 2.2. Extraction of Phospholipids from Camel Milk

Extraction of the whole lipid fraction was carried out from 500 mL of camel milk. The freeze drying technique was applied to avoid milk and water interference, as well as the interaction of phospholipids with both lipids and proteins, due to their amphiphilic properties [22]. The freeze-dried milk sample was extracted three times with methylene chloride (chloroform was replaced with methylene chloride due to toxicity). The residue was then extracted with a mixture of 1 : 1 (v/v) methylene chloride/methanol mixture and finally with 100% methanol. After evaporating the solvent mixtures, the lipid content was evaluated by thin layer chromatography (TLC) using methylene chloride : methanol : acetic acid : water (65 : 30 : 6 : 2) as developing system. The combined methylene chloride fraction was evaporated under vacuum, and the finally dried residue was subjected to vacuum liquid chromatography (VLC) using methylene chloride for neutral lipids and methanol for phospholipids [23]. The purified phospholipids were primarily evaluated by TLC and compared with standard phospholipids. Identification of individual phospholipids was achieved by GC-MS and HPLC.

### 2.3. HPLC System and Conditions

The analytical HPLC system consisted of an Alliance 1525 separation module, automatic HPLC system equipped with UV/Vis detector 2489 (Waters, USA). The separation was achieved on Spherisorb C8 column (4.6 × 250 mm; particle size 5 *μ*m) at 45°C. The mobile phase consisted of A: acetonitrile/methanol/n-hexane (90 : 3 : 7 v/v) with 0.08% triethyamine (TEA) and B: acetonitrile/water/phosphoric acid (75 : 25 : 1 v/v) with 0.08% TEA. The rate of flow was 1-2 mL/min with an injection volume of 20 *μ*L. The monitoring wavelength was 210 nm.

### 2.4. GC/MS System and Conditions

The GC-MS analysis was performed in a Shimadzu GC 2014 series equipped with a flame ionization detector. A DB-35 capillary column (30 mm × 0.25 mm × 25 *μ*m). The initial temperature was 80°C for 4 min after injection, increased to 280°C with a final hold at 280°C for 25 min. The injector and detector temperature were maintained at 270°C. Helium was used as a carrier gas at a flow rate of 0.7 mL/min.

### 2.5. Liposome Preparation and Drug Encapsulation

Liposomes from DPPC and camel milk phospholipids containing etoposide were prepared. The thin film hydration method was used. Briefly, liposomes from DPPC or camel milk phospholipids were prepared by dissolving the lipid and etoposide in a mixture of chloroform/methanol. The solvent was subsequently slowly evaporated in a rotary evaporator. Multilamellar large vesicles (MLVs) were formed by adding normal saline. The preparation was then treated by freeze-thaw for 10 cycles. The so-produced vesicles were subsequently extruded 10 times through 200 nm and 100 nm polycarbonate membranes using an extruder device Lipex Biomembranes Inc., heated at 50°C. The final lipid concentration of the formulations was 10 mg/mL. Subsequently, the extruded vesicles with encapsulated etoposide were separated from unentrapped etoposide by filtration through a Sephadex G-75 column. Vesicles were disrupted with ethanol and the entrapped etoposide was assayed by HPLC method as described above.

### 2.6. Characterization of Liposomes

An optical microscope (SZII, Olympus, Tokyo, Japan) equipped with a CCD camera (SSC-DC50A, SONY, Tokyo, Japan) was used to take microphotographs for Cam-liposomes. The size distribution for the DPPC-liposomes and Cam-liposomes (liposomes from camel milk phospholipids) before and after extrusion was determined using the Mastersizer by Malvern Instruments Ltd.

### 2.7. Benzo(a)pyrene- (BAP-) Induced Tumors in a Mouse Model

Female BALB/c mice (6–8 weeks of age) were used to induce tumors in mice. Tumors were induced chemically by injecting benzo(a)pyrene (BAP) dissolved in sesame oil at a dose of 250 *μ*g/mouse through subcutaneous route as described earlier [24]. After 90–120 days after BAP injections mice developed palpable tumors.

### 2.8. Treatment of Tumor-Bearing Mice

Tumor-bearing mice were divided into six different treatment groups and each group was comprised of 10 mice:

1. untreated control,
2. Sham DPPC-liposomes,
3. Sham camel milk liposomes,
4. free etoposide (F-ETP),
5. etoposide-loaded DPPC-liposomes (ETP-DPPC-liposomes),
6. etoposide-loaded camel milk liposomes (ETP-Cam-liposomes).

Tumors were measured regularly using vernier caliper until they reach the volume of 200 mm^3^. At this point, the treatment of mice was started with drug formulations. Various formulations of etoposide at the dose of 5 mg/kg were administered intraperitoneally to treat tumor-bearing mice once a week for three weeks. The first day of treatment was considered day zero. The size of the tumors was measured regularly according to following formula:

(1) $$\begin{array}{c} V=D\times d2\times \frac{\pi }{6}, \end{array}$$

where *V* = tumor volume, *D* = biggest dimension, and *d* = smallest dimension.

The efficacy of the treatment was assessed by analyzing the survival and measuring the change in tumor size of the treated mice.

### 2.9. Statistics

Survival of the mice was analyzed by log-rank test using Kaplan-Meier curve. The sizes of tumors were compared by one-way ANOVA using Prism software version 6.0 (San Diego, USA).

## 3. Results and Discussion

### 3.1. Identification of Major Lipid Components in Camel Milk

The main aim of the present study is not to identify and characterize the phospholipids composition of the camel milk as it has been characterized by many researchers earlier [25, 26]. We characterized the phospholipid composition of camel milk to get the general idea of the presence of the major phospholipids. The TLC plates revealed the presence of PE, PC, LPC, and PI as major phospholipids. Identification of individual phospholipids was achieved by GC-MS and HPLC. It is important to note that interference in the UV/Vis spectrophotometer between etoposide and camel milk phospholipids was observed. This interference necessitated the development of a new and rapid HPLC method not only for phospholipid identification, but also for determination of the encapsulated etoposide into liposomes composed of camel milk phospholipids.

Several mobile phase solvent systems (including isocratic and gradient mobile phase) were, respectively, tested. Their chromatography behavior was simultaneously compared. It was found that gradient mobile phase containing A: acetonitrile/methanol/n-hexane (90 : 3 : 7 v/v) with 0.08% triethyamine (TEA) and B: acetonitrile/water/phosphoric acid (75 : 24 : 1 v/v) with 0.08% TEA and a flow rate of 1-2 mL/min was successful in separating the four major PLs. These conditions were used to measure the calibration curves of individual phospholipids standards.

**Figure 1:**
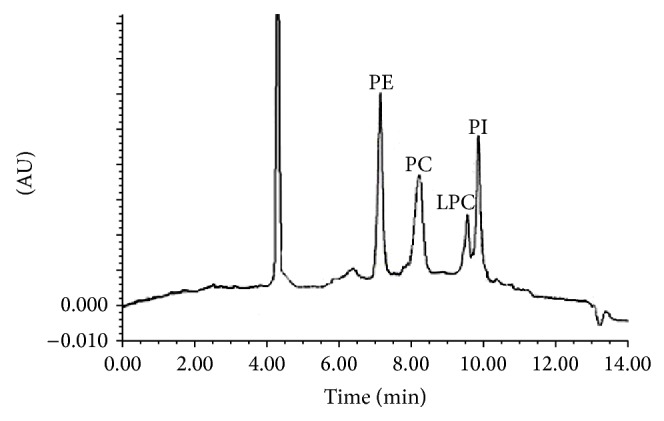
Chromatogram of standard mixture of phospholipids with UV-detection at 210 nm.

Because of the lipophilicity of the phospholipids, we tried to separate them on a Spherisorb C8 column. The investigated phospholipids are varied in their chemical characteristics so we use phosphoric acid to ensure the elution of all the four analytes, to neutralize the charged phosphate group in phospholipids and to reduce secondary interactions between the polar functionality of the phospholipids and any acidic silanols on the stationary phase, which results in strong retention and peak tailing. Under the above-described conditions, the complete separation of the four investigated phospholipids was achieved in less than 12 min. (Figure 1). It shows a typical chromatogram obtained from a standard mixture of PC, PE, PI, and LPC at 210 nm. Peak shapes were improved by both the addition of phosphoric acid and TEA and increasing the temperature. When the temperature of the assay was stable (45°C), the peak appearance and retention times were highly reproducible.

### 3.2. Validation of the Method

#### 3.2.1. Linearity, Detection, and Quantitative Limits

A linear relationship between the concentrations of PC, PE, PI, and LPC and the UV absorbance at 210 nm was obtained. This linearity was maintained over the concentration ranges of 5–64 *μ*g mL^−1^ for PC, 10–250 *μ*g mL^−1^ for PE, 15–160 *μ*g mL^−1^ for PI, and 50–160 *μ*g mL^−1^ for LPC. The correlation coefficient for each standard curve invariably exceeded 0.997. The values of the detection limit were calculated and were 0.489, 3.05, 4.5, and 9.52 *μ*g mL^−1^ for PC, PE, PI, and LPC, respectively. The results were summarized in Table 1. The assay exhibits the necessary sensitivity and linearity to cover the physiological concentrations of the PLs in milk.

### 3.3. Precision

Intraday and interday precision were assessed using three concentrations and three replicates of each concentration. The calculated relative standard deviation values were found in Table 2. All the values are acceptable for bioanalytical methods.

### 3.4. Accuracy

Accuracy was also assessed by the recovery of the added standard; three concentrations each in triplicate to a known concentration of camel milk sample were tested by the proposed method. HPLC conditions were as follows: mobile phase A: acetonitrile/methanol/n-hexane (90 : 3 : 7 v/v) with 0.08% triethyamine (TEA) and B: acetonitrile/water/phosphoric acid (75 : 25 : 1 v/v) with 0.08% TEA and a flow rate of 1-2 mL/min; column temperature, 45°C; and UV at 210 nm.

### 3.5. Recovery

The recovery of the method was evaluated by comparing the response of extracted camel milk samples spiked before extraction with the response of extracted blank samples spiked just before injection.  Table 3 shows the results of recoveries. All those recoveries are between 92.41 and 96.74 and RSDs are less than 2%, indicating good accuracy of the recovery of the method.

The scope of the protocol was to choose a mobile phase that provides a good selectivity with no too large cutoff of absorbance at a wavelength below 210 nm. These conditions were met by using a mixture of A: acetonitrile/methanol/n-hexane (90 : 3 : 7 v/v) with 0.08% triethyamine (TEA) and B: acetonitrile/water/phosphoric acid (75 : 25 : 1 v/v) with 0.08% TEA. The procedure was applied to determine PE, PC, PI, and LPC in camel milk samples. The same method was also used for etoposide determination into liposomes prepared from camel milk phospholipids. The quantitative determination was carried out using a simple calibration procedure. GC/MS was used to confirm the structure of the separated PLs. On the whole, it is interesting to observe that the proposed HPLC procedure offers the advantage of its simplicity in the detection of phospholipids used in this study by using a simple UV detector.

### 3.6. Gas Chromatography-Mass Spectrometry (GC/MS)

The phospholipid fractions were subjected to capillary gas chromatography-mass spectrometry for profiling the phospholipid content in a total run of 45 minutes and full mass spectrum between 100 and 1000 was obtained. The main phospholipid constituents based on their characteristic mass fragments were PE, PC, PI, and LPC.

**Figure 2:**
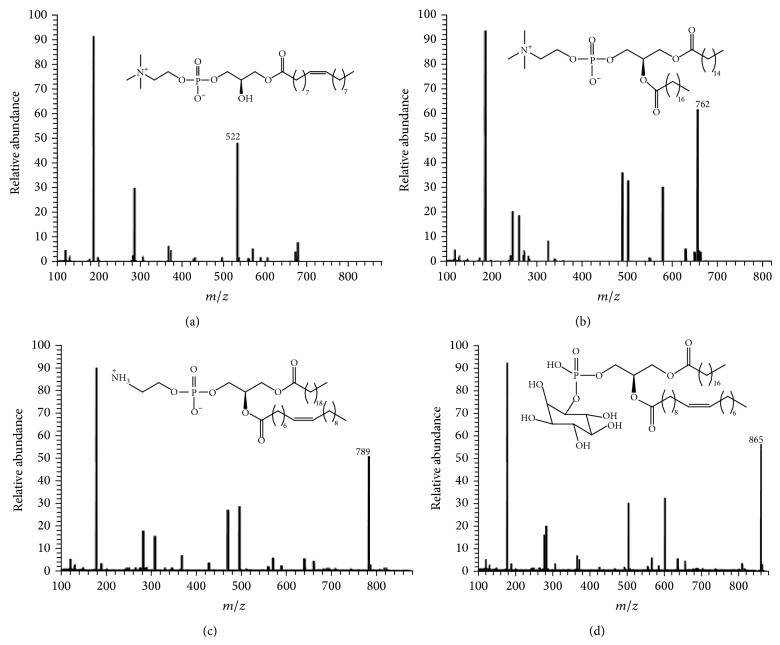
MS spectrograms of (a) LPC, (b) PC, (c) PE, and (d) PL.

Lysophosphatidylcholine (LPC) showed the quasimolecular ion peak at* m/z* 522 [M + H]^+^ assigned to the protonated molecule and base peak at* m/z* 183 corresponding to phosphorylcholine [PO_4_(CH_2_)_2_N(CH_3_)_3_ + H]^+^ moiety. The ion peak at* m/z* 282 [FA + H]^+^ revealed octadecenoic fatty acid residue (18 : 1). Accordingly, the phospholipid species were assigned octadecenoic 18 : 1 LPC (Figure 2(a)).

Phosphatidylcholine (PC) was recognized from quasimolecular ion peak at* m/z* 761 [M + H]^+^, the characteristic phosphorylcholine base peak at* m/z* 183 [PO_4_(CH_2_)_2_N(CH_3_)_3_ + H]^+^, and low abundance peaks at* m/z* 580 assigned to [M–phosphorylcholine]^+^. The ion peaks at* m/z* 494, 268, and* m/z* 506, 256 were assigned to the loss of octadecanoic 18 : 0 and hexadecanoic 16 : 0 fatty acid moieties, respectively, which identify this species as 18 : 0/16 : 0 diacyl PC (Figure 2(b)).

Phosphatidylethanolamine (PE) provided an ion peak at* m/z* 181 characterizing the loss of phosphoethanolamine moiety. The quasimolecular ion peak at* m/z* 789 [M + H]^+^, the ion peaks at* m/z* 461, 312 and at* m/z* 491, 282 were assigned to eicosanoic 20 : 0 and octadecenoic 18 : 1 acids, respectively, which identified this phospholipid species as 20 : 0/18 : 1 diacyl PE (Figure 2(c)).

Phosphatidylinositol (PI), the negative mode ionization spectrum, showed an ion peak at* m/z *864 [M + H]^+^ and an ion peak at* m/z* 180 characterizing the loss of an inositol moiety.

The ion peak at* m/z *581 [M + R_1_]^+^ together with the peak at* m*/*z* 283 represents the losses of octadecanoyl 18 : 0, and the peak at* m*/*z* 583 [M + R_2_]^+^ together with the peak 281 represents the losses of octadece-9-noyl 18 : 1,* m/z *281, which identified this phospholipid species as 18 : 0/18 : 1 diacyl PI (Figure 2(d)).

### 3.7. Liposome Preparation and Drug Encapsulation

**Figure 3:**
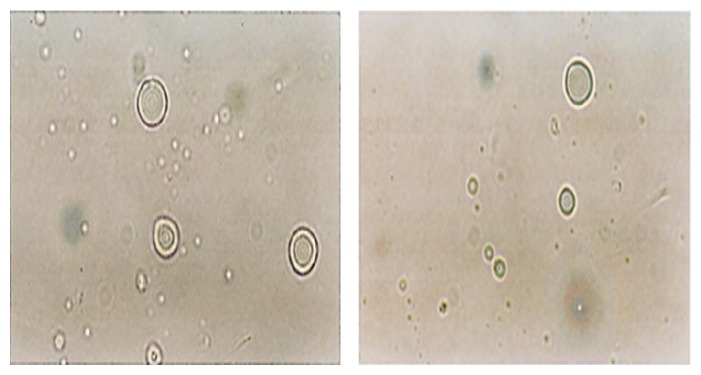
Microphotographs for liposomes prepared from camel milk phospholipids.

**Figure 4:**
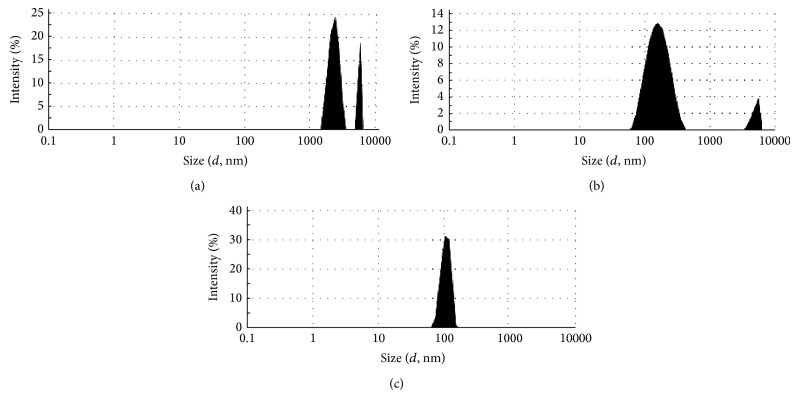
Size distribution for liposomes prepared from DPPC. (a) MLVs liposomes before extrusion, (b) liposomes after extrusion 10 times through 200 nm polycarbonate membrane, and (c) SUVs liposomes after extrusion 10 times through 100 nm polycarbonate membrane.

**Figure 5:**
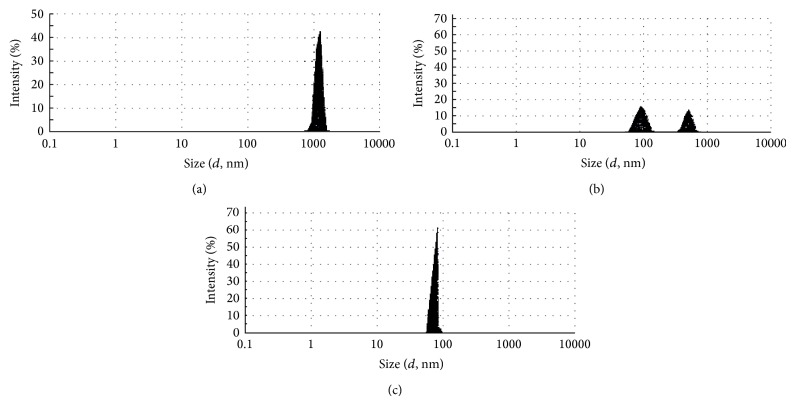
Size distribution for liposomes prepared from camel milk phospholipids. (a) MLVs liposomes before extrusion, (b) liposomes after extrusion 10 times through 200 nm polycarbonate membrane, and (c) SUVs liposomes after extrusion 10 times through 100 nm polycarbonate membrane.

Microphotographs obtained from the optical microscopy show that MLVs liposomes were successfully prepared from a mixture of phospholipid isolated from camel milk by using the thin film hydration method (Figure 3). The vesicle size distributions for DPPC-liposomes and Cam-liposomes before and after the extrusion through 200 nm followed by 100 nm polycarbonate membrane (10 times for each) were determined using a Malvern Mastersizer. Figures 4 and 5 show that the repetitive extrusion of MLVs through the stacked polycarbonate membrane with 200 nm followed by 100 nm pore size after 10 freeze-thaw cycles resulted in small unilamellar vesicles (SUVs) exhibiting a relatively homogeneous size distribution. The size distribution for DPPC-liposomes before extrusion was 2312 nm for the first peak with 80.65% intensity and 5557 nm for the second peak with 19.4% intensity (Figure 4(a)). The size distribution after 10-time extrusions through 200 nm polycarbonate membrane was 181.3 nm with 91.5% intensity for the first peak and 5000 nm with 8.5% intensity for the second peak (Figure 4(b)). While after 10-time extrusions through 100 nm polycarbonate membrane, only one peak was obtained at 113 nm with 100% intensity (Figure 4(c)).

The size of Cam-liposomes after hydration (before extrusion) was smaller than DPPC-liposomes with only one size distribution peak at 1242 nm with 100% intensity (Figure 5(a)). Also Cam-liposomes were smaller after 10-time extrusions through 200 nm polycarbonate membrane with size distribution at 95.2 nm for the first peak and 532.1 nm for the second peak (Figure 5(b)). After 10-time extrusions of Cam-liposomes through 100 nm polycarbonate membrane, the size distribution was 75.75 nm with 100% intensity (Figure 5(c)). It is important to note that the size distribution of Cam-liposomes indicates that Cam-liposomes were smaller than that for DPPC-liposomes before and after extrusion.

Etoposide was encapsulated into DPPC-liposomes with the trapping efficiency of not more than 18%, while the percent of etoposide encapsulation was increased in Cam-liposomes to 22%. The increase in the % trapping efficiency of etoposide into Cam-liposomes may be due to their phospholipid composition.

### 3.8. In Vivo Activity of Various Etoposide Formulations against Experimental Fibrosarcoma

**Figure 6:**
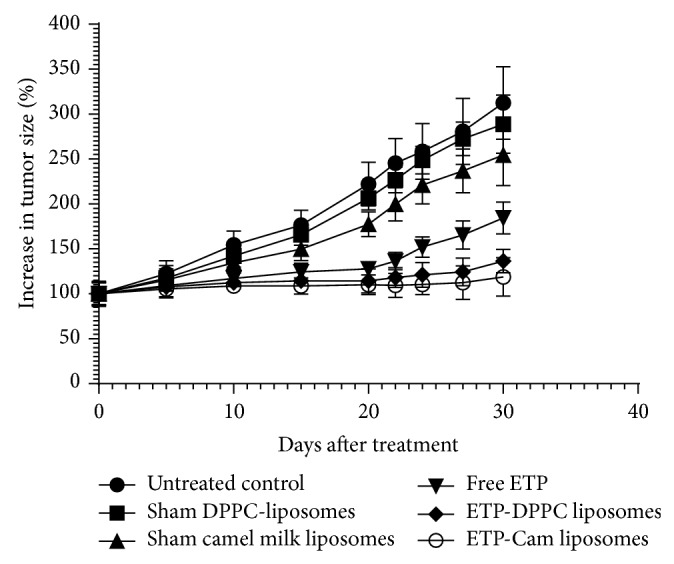
Effects of various formulations of etoposide on benzo(a)pyrene- (BAP-) induced tumors in a mouse model. Treatment of tumor-bearing animals was started when the tumor size reached a volume of approximately 200 mm^3^. Mice treated with ETP-DPPC-liposomes or ETP-Cam-liposomes showed delayed tumor growth (*P* < 0.05) as compared with controls (PBS and Sham liposomes). Treatment with ETP-DPPC-liposomes or ETP-Cam-liposomes was superior to free ETP (*P* < 0.001). Data are values ± SD (*n* = 10 at initiation of therapy; the number varies at later time points due to mortality).

**Figure 7:**
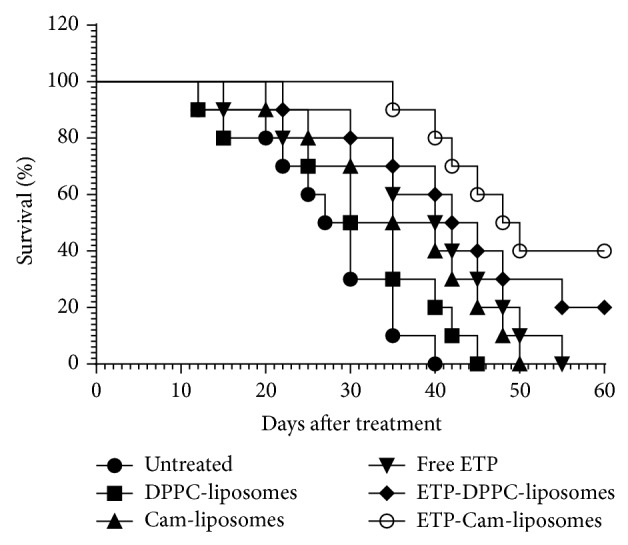
Effects of various formulations of ETP chemotherapy on the survival of tumor-bearing mice. The fibrosarcoma was induced by exposure to benzo(a)pyrene. The treatment of tumor-bearing animals was started at the time point when tumor size reached a volume of approximately 200 mm^3^. Various formulations of etoposide at the dose of 5 mg/kg were administered intraperitoneally to treat tumor-bearing mice twice weekly for three weeks. The first day of treatment was considered day zero. Free ETP versus untreated control (*P* = 0.0077); ETP-DPPC-liposomes versus free ETP (*P* = 0.2838); and ETP-Cam-liposomes versus free ETP (*P* = 0.0303).

The* in vivo* activity of various etoposide formulations against experimental fibrosarcoma was assessed in a murine model. Chemotherapy was started when tumors attained the size in the range of 150–200 mm^3^. Fibrosarcoma-bearing mice were treated with a single weekly dose of 5 mg/kg of free ETP or ETP-DPPC-liposomes or ETP-Cam-liposomes through intraperitoneal route for three weeks. During and after the chemotherapy the size of the tumors was regularly measured using digital vernier calipers. Among the all treatment groups, the tumors showed the least progression or delayed growth in mice treated with ETP-Cam-liposomes, whereas the tumors in mice treated with free ETP showed more growth compared to those treated with ETP-DPPC-liposomes or ETP-Cam-liposomes (Figure 6). However, it is important to note that once the chemotherapy was stopped, tumor size started to increase very fast. Most of the mice in group treated with ETP-Cam-liposomes died in between 40 and 60 days after treatment (Figure 7).

Besides assessing the effect of chemotherapy on the size of tumors, the effect of various formulations of etoposide on the survival of tumor-bearing mice was also observed. Consistent with its effect in restricting tumor size, chemotherapy with ETP-Cam-liposomes also imparted greater survival to fibrosarcoma-bearing mice. Tumor-bearing mice treated with ETP-Cam-liposomes died within 120 days, whereas mice in the groups treated with free ETP or ETP-DPPC-liposomes died within 70 days and 90 days, respectively (Figure 7).

There have been many beneficial effects associated with the use of camel milk in human beings [27–29]. Camel milk has been shown to possess therapeutic value in the treatment of cancer [30–32]. Most of the beneficial effects are associated with the protein components present in camel milk. But the role of phospholipid components of camel milk has not been explored. In the present study, we exploited the camel milk phospholipids for the preparation of liposomes and used them as drug delivery systems for anticancer drug etoposide against fibrosarcoma in a murine model. The characterization of the phospholipids isolated from camel milk predominantly showed the presence of PE, PC, PI, and LPC. Camel milk contains interesting lipid composition as characterized by the different methods used in the present study. The presence of phosphatidylethanolamine (PE) in camel milk phospholipid liposomes may play important role in increasing the anticancer efficacy of etoposide. Earlier studies have shown the increased efficacy of anticancer drug cisplatin entrapped in PE-containing liposomes [33]. This is also confirmed by the results of the present study that showed greater efficacy of ETP-Cam-liposomes compared to the ETP-DPPC-liposomes or free ETP. Commonly used cationic liposomes also show greater toxicity against normal cells, whereas the presence of PE imparts a negative charge to the liposomes and reduces their toxicity to normal cells.

## 4. Conclusions

This study tests a chromatographic protocol for the separation and qualitative/quantitative determination of four PLs in camel milk by HPLC with a UV detector. The procedure was applied for the determination of the PE, PC, PI, and LPC in camel milk. This is the first study in which camel milk phospholipids were used to prepare liposomes to deliver anticancer drugs. The results of the present study showed that etoposide entrapped in camel milk phospholipid liposomes shows greater anticancer activity against fibrosarcoma in a murine model compared to free etoposide or etoposide entrapped in DPPC-liposomes. This suggests that camel milk phospholipids may prove to be better and more effective delivery systems for anticancer drugs in human beings.

## Figures and Tables

**Table 1:** Analytical parameters calculated for each standard phospholipid.

Parameter	PC	PE	PI	LPC
Linear range (*μ*g mL^−1^)	5–64	10–250	15–160	50–160
Intercept (*a*)	18.77	65.51	196.94	−23.06
SE of intercept (*S*_*a*_)	6.78	60.55	121.08	8.17
Slope (*b*)	45.75	67.63	89.71	2.83
SE of slope (*S*_*b*_)	0.175	0.537	1.32	0.07
Correlation coefficient (*r*)	0.9999	0.9998	0.9991	0.9988
Determination coefficient (*r* ^2^)	0.9999	0.9998	0.9982	0.9975
LOD (*μ*g mL^−1^)	0.489	3.05	4.5	9.52
LOQ (*μ*g mL^−1^)	1.48	9.24	13.64	28.86

**Table 2:** Intraday and interday precision of the proposed method.

Parameter		PC			PE			PI			LPC		
		10 *µ*g mL^−1^	30 *µ*g mL^−1^	50 *µ*g mL^−1^	20 *µ*g mL^−1^	80 *µ*g mL^−1^	120 *µ*g mL^−1^	20 *µ*g mL^−1^	50 *µ*g mL^−1^	100 *µ*g mL^−1^	60 *µ*g mL^−1^	90 *µ*g mL^−1^	120 *µ*g mL^−1^
Intraday	1	101.07	101.74	100.89	98.25	97.7	103.91	97.12	96.37	96.61	90.00	87.50	96.51
	2	101.80	102.65	101.08	99.01	101.54	101.1	99.51	10024	94.43	91.25	89.50	99.63
	3	93.98	104.94	102.99	102.13	99.25	99.44	96.91	99.11	97.42	91.50	86.25	98.1
	Mean	**100.61**	**103.11**	**101.65**	**99.79**	**99.49**	**101.48**	**97.84**	**98.57**	**96.15**	**90.91**	**87.75**	**98.08**
	S.D	**1.46**	**1.64**	**1.16**	**2.05**	**1.93**	**2.25**	**1.44**	**1.99**	**1.54**	**0.80**	**1.63**	**1.56**
	R.S.D	**1.45**	**1.59**	**1.14**	**2.05**	**1.93**	**2.21**	**1.47**	**2.01**	**1.59**	**0.87**	**1.85**	**1.59**

Interday	1	99.63	101.03	102.65	100.56	99.33	101.19	95.37	96.61	99.11	98.85	98.9	96.98
	2	99.83	101.27	99.82	99.82	98.86	100.17	99.22	100.80	98.94	101.5	99.25	98.99
	3	101.4	96.39	103.88	98.33	95.63	103.22	98.18	99.88	99.75	97.76	101.00	97.56
	Mean	**100.28**	**99.56**	**102.11**	**99.57**	**97.94**	**101.52**	**97.59**	**99.09**	**99.26**	**99.37**	**99.71**	**97.84**
	S.D	**0.969**	**2.75**	**2.08**	**1.13**	**2.01**	**1.55**	**1.99**	**2.20**	**0.42**	**1.92**	**1.12**	**1.03**
	R.S.D	**0.966**	**2.76**	**2.05**	**1.13**	**2.05**	**1.52**	**2.03**	**2.22**	**0.43**	**1.93**	**1.12**	**1.05**

**Table 3:** Results of recovery studies and their relative standard deviations.

Sample number	PC			PE			PI			LPC		
	Taken (*µ*g mL^−1^)	Found (*µ*g mL^−1^)	% of recovery	Taken (*µ*g mL^−1^)	Found (*µ*g mL^−1^)	% of recovery	Taken (*µ*g mL^−1^)	Found (*µ*g mL^−1^)	% of recovery	Taken (*µ*g mL^−1^)	Found (*µ*g mL^−1^)	% of recovery
1	10	9.55	95.53	20	19.34	96.74	20	18.62	93.14	60	57.00	95.01
2	30	28.39	94.64	80	74.43	93.04	50	46.87	93.75	90	84.05	93.39
3	50	46.20	92.41	120	114.6	95.5	120	114.98	95.82	120	115.27	96.06
Mean			94.19			95.09			94.23			94.82
S.D.			1.60			1.88			1.40			1.34
R.S.D.			1.69			1.97			1.48			1.41
